# *In silico* mining of putative microsatellite markers from whole genome sequence of water buffalo (*Bubalus bubalis*) and development of first BuffSatDB

**DOI:** 10.1186/1471-2164-14-43

**Published:** 2013-01-19

**Authors:** Sarika, Vasu Arora, Mir Asif Iquebal, Anil Rai, Dinesh Kumar

**Affiliations:** 1 Centre for Agricultural Bioinformatics, Indian Agricultural Statistics Research Institute, Library Avenue, New Delhi, 110012, India; 2 Division of Biometrics & Statistical Modelling, Indian Agricultural Statistics Research Institute, Library Avenue, New Delhi, 110012, India; 3 Genes & Genetic Resources Molecular Analysis Lab, National Bureau of Animal Genetic Resources, Karnal, Haryana, 132001, India

**Keywords:** *de novo*, Microsatellites, Primers, Radiation hybrid, Water buffalo

## Abstract

**Background:**

Though India has sequenced water buffalo genome but its draft assembly is based on cattle genome *BTau 4.0*, thus *de novo* chromosome wise assembly is a major pending issue for global community. The existing radiation hybrid of buffalo and these reported STR can be used further in final gap plugging and “finishing” expected in *de novo* genome assembly. QTL and gene mapping needs mining of putative STR from buffalo genome at equal interval on each and every chromosome. Such markers have potential role in improvement of desirable characteristics, such as high milk yields, resistance to diseases, high growth rate. The STR mining from whole genome and development of user friendly database is yet to be done to reap the benefit of whole genome sequence.

**Description:**

By *in silico* microsatellite mining of whole genome, we have developed first STR database of water buffalo, *BuffSatDb* (Buffalo MicroSatellite Database (http://cabindb.iasri.res.in/buffsatdb/) which is a web based relational database of 910529 microsatellite markers, developed using PHP and MySQL database. Microsatellite markers have been generated using MIcroSAtellite tool. It is simple and systematic web based search for customised retrieval of chromosome wise and genome-wide microsatellites. Search has been enabled based on chromosomes, motif type (mono-hexa), repeat motif and repeat kind (simple and composite). The search may be customised by limiting location of STR on chromosome as well as number of markers in that range. This is a novel approach and not been implemented in any of the existing marker database. This database has been further appended with Primer3 for primer designing of the selected markers enabling researcher to select markers of choice at desired interval over the chromosome. The unique add-on of degenerate bases further helps in resolving presence of degenerate bases in current buffalo assembly.

**Conclusion:**

Being first buffalo STR database in the world , this would not only pave the way in resolving current assembly problem but shall be of immense use for global community in QTL/gene mapping critically required to increase knowledge in the endeavour to increase buffalo productivity, especially for third world country where rural economy is significantly dependent on buffalo productivity.

## Background

Water buffalo (*Bubalus bubalis*) contributes immensely to the agricultural economy of Indian subcontinent, South East Asian countries through milk, meat, hides, fertilizer, fuel and draught animal power. A large part of human population depends on this species than any other livestock species in the world [1]. There is 188.3 million buffalo population in the world which contributes around 55 – 60% of total milk production [2]. Asia has nearly 97% of buffaloes and is an integral part of agriculture in India, China, Pakistan, Nepal, Bangladesh, Thailand, Myanmar and Malaysia. The productivity of buffaloes in these regions is higher as compared to cattle [3].

Molecular markers can play a significant role for livestock improvement through conventional breeding strategies. Scientific resources are limited in many of the countries where buffaloes are economically important livestock and as a consequence, genome research has not been supported at the level of some of the other species [4]. Limited number of researches has been conducted globally exploring the genetic diversity on molecular genetic basis in buffalo in comparison with other farm animal genetic resources. This depends, in part on the knowledge of their genetic structure based on molecular markers like microsatellites [5].

Microsatellites are sequences made up of a simple sequence motif, not more than six bases long, that is tandemly repeated and arranged head to tail without interruption by any other base or motif. Simple, tandemly repeated di- and tri- nucleotide sequences have been demonstrated to be polymorphic in length in a number of eukaryotic genome [6]. The frequency with which they occur (once every 50,000–60,000 bp), the high degree of polymorphism displayed, and their random distribution across the genome [7] make them potentially very useful as DNA markers in gene mapping studies. Furthermore, two or more microsatellites may be analyzed simultaneously [8,9], opening new opportunity for genetic analysis of large number of samples.

To cater the need of microsatellite especially for biodiversity analysis, cattle microsatellite markers have been used in heterologous mode in buffalo and up to 56% of them have been found polymorphic [10]. Cattle microsatellite markers have many disadvantages in such diversity analysis like low polymorphism and loss of amplification due to null alleles, size biasness, hitch hiking and potential exclusion of abundant STR in gene pool [11]. Even there is limited work of STR mining using partial enriched genomic library [12].

There is no thorough *in silico* STR marker mining from buffalo genome to represent more holistic and cumulative variability of genome to be used in gene pool or biodiversity analysis and gene/QTL mapping. Though India has sequenced water buffalo genome but its draft assembly is based on cattle genome *BTau 4.0*, thus *de novo* chromosome wise assembly is a major pending issue for global community [3]. The existing radiation hybrid of buffalo by Amaral *et al.*[13] and these reported STR can be used further in final gap plugging and “finishing” expected in *de novo* genome assembly. Such work needs extensive STR mining from buffalo genome at equal interval on each and every chromosome. In order to cater this urgent need in resolving assembly, mapping issues and biodiversity analysis, we have developed first STR database of water buffalo, *BuffSatDb* (Buffalo MicroSatellite Database) which is a web based relational database of microsatellites.

## Construction and content

### Data collection and architecture

**Figure 1:**
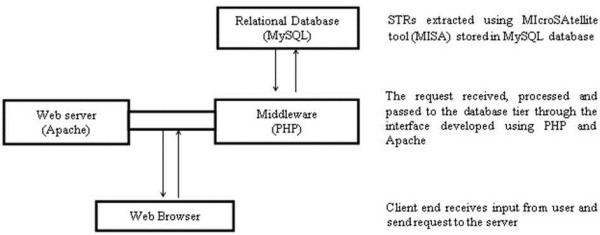
**Three-tier architecture of *BuffSatDB*.
**

The *BuffSatDb* is an online relational database that catalogues information about the microsatellite repeats of the recently sequenced water buffalo. All the microsatellite markers extracted from buffalo genome have been generated using MIcroSAtellite tool (MISA) [14]. The database architecture is a “Three-tier architecture” (Figure 1) with a client tier, middle tier and database tier. This user-friendly interface for the database has been developed using PHP (**H**ypertext **P**reprocessor) which is an open-source server-side scripting language. In first tier of the architecture, the *in silico* mined STRs through MISA were stored in MySQL database. In the middleware, user need based customised query provisions have been made. For primer designing, Primer3 standalone code computes primers on user request. The information generated at the client end, i.e. third tier of the architecture are list of multiple primers along with their respective melting temperature, GC content, start position and product size (amplicon size).

*BuffSatDb* has eight tabs (Home, About, Database, Analysis, Tutorial, Links, Contact, Team). General information of the developed microsatellite database, information about Water buffalo, microsatellite markers, comparative analysis of the buffalo genome has been discussed. The tutorial of this database contains the guidelines for users and terminologies used in the database contents. *BuffSatDb* is appended with other useful links, the team and contact persons.

### In silico mining of microsatellite from whole genome of water buffalo

**Figure 2:**
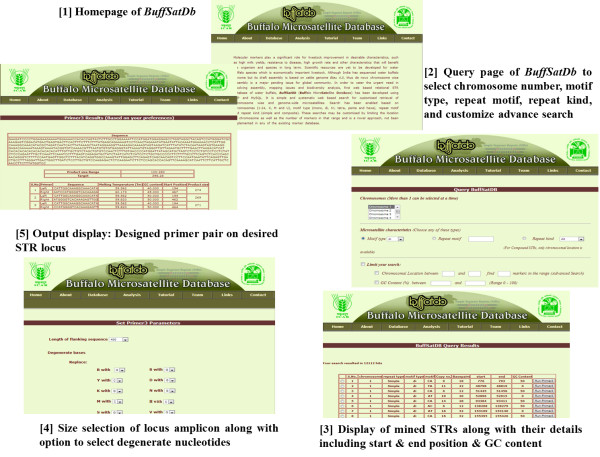
**The flow of database search in *BuffSatDb*.
**

The *Bubalus bubalis* genome draft assembly version Bbu_2.0-alpha which is with 17X-19X depth and 91%–95% coverage published by research group from India [3] and available in public domain at http://210.212.93.84/bbu_2.0alpha/ was used for STR mining. All the 27 available chromosomes (Chromosome 1–24, M, U and X) were chopped into manageable range using PERL script. These were fed to MIcroSAtellite identification tool, MISA to identify and find the location of perfect and compound microsatellites. The STR numbers, motifs, repeat number, length of the repeat, size of the repeat, repeat type, GC content, start and end position of the repeat and STR sequence were compiled. A total of 910529 STRs were generated from water buffalo genome, of which 830058 were simple and 80471 were compound STRs. *BuffSatDb* is the comprehensive and integrated resource for retrieval of information from water buffalo. Figure 2 shows the database search in *BuffSatDb*.

The user can query for microsatellites, chromosome wise (1–24, X, M and U), where more than one chromosome may be selected at a time from water buffalo genome. These searches may further be customised based on microsatellite characteristics like motif type (mono, di, tri, tetra, penta, hexa), repeat motif and repeat kind (simple and composite). The user may further go for advance search like limiting the location on chromosome as well as the number of markers in that range. This is a novel approach and to the best of our knowledge, it has not been implemented in any of the existing marker database which may be useful for the researchers. Identification of QTL and fine mapping of economically important genes based on LOD (Logarithm of the Odds) score also needs STR preferably at equal interval. Also other parameters like GC content, range of STR location and copy number may be customised for the above selection according to the requirement of researchers. The results are then displayed in tabular format, giving chromosome number, motif type, motif, copy number, basepair, start and end position along with the GC content.

*BuffSatDb* is further appended with Primer3 tool [15]. The STRs traced by the query, may be selected with the help of radiobutton for generation of primers. Primer for selected STR locus may be designed with a template of approximately 1000 base pairs by selecting upto 500 base pairs of both flanking regions. These flexibilities would enable researchers to select markers of choice at desired interval over the chromosomes. Further one can use each individual STR of a targeted region over chromosome to narrow down location of gene of interest or linked QTL. A novel add-on for degenerate bases has been incorporated in this database search, where the users are given flexibility to replace degenerate bases with any of the alternative bases (A,T,G,C). This feature has been added to resolve the issue of some of the degenerate bases present in current buffalo genome assembly making the primer designing very difficult otherwise.

### Genome analysis

**Figure 3:**
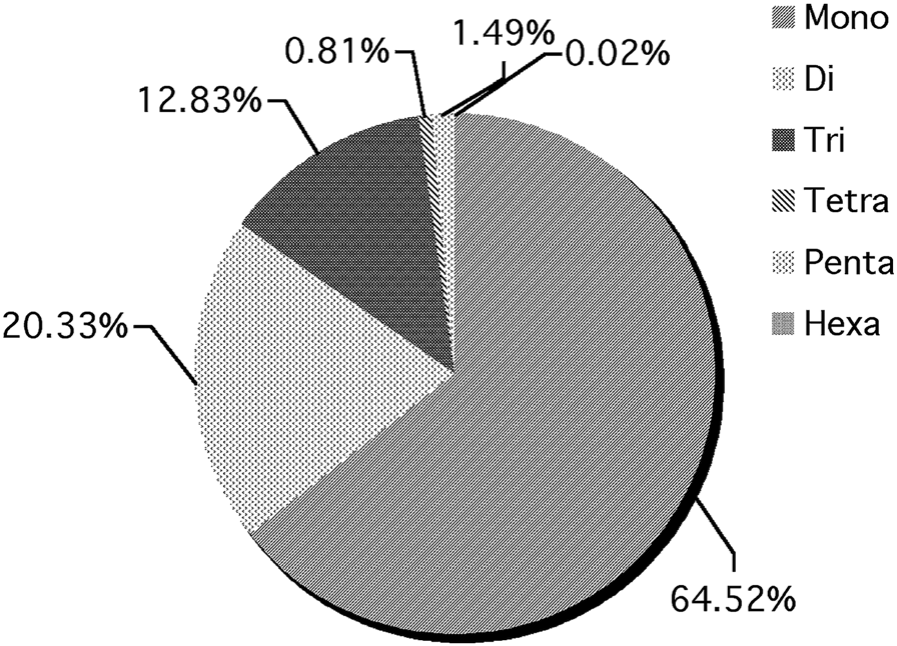
Graphical view of motif wise distribution of microsatellites in Buffalo genome.

**Figure 4:**
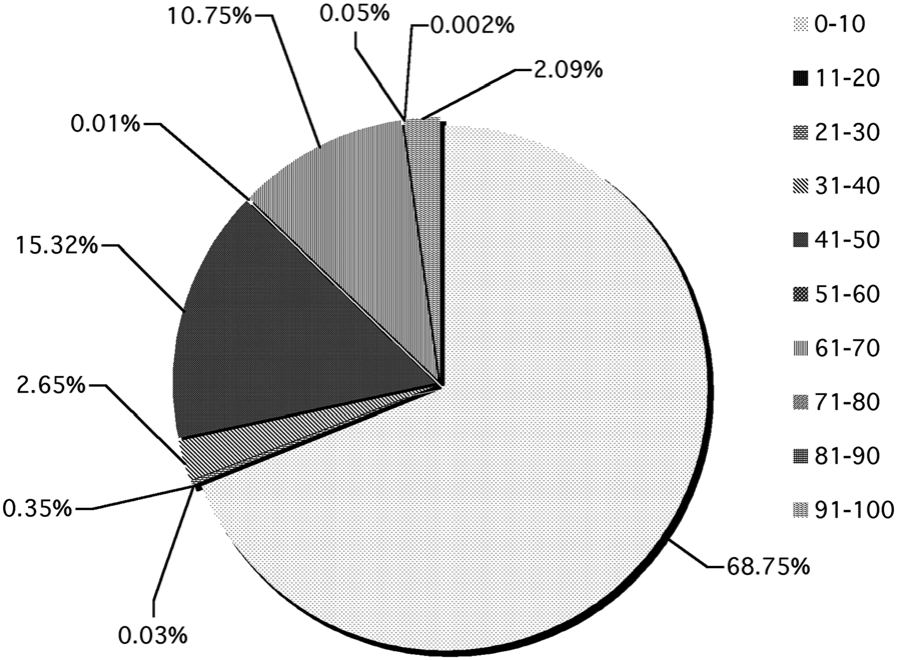
Graphical view of proportion of GC content in STRs at various ranges.

**Figure 5:**
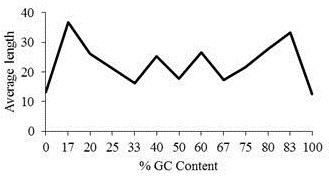
Distribution of length of microsatellites in context to GC percentage.

The chromosome wise distribution of STRs along with its respective motif frequencies in buffalo genome were analysed. It was observed that simple STRs constituted most abundantly with 91.16% of the total STRs. Various motif types like, mono, di, tri, tetra, penta and hexa type of microsatellites have been plotted to show the respective abundance of the type in chromosomes. Mono type (64.52%) was seen to have abundance than any other types while the hexa (0.02) was the one with least occurrence (Figure 3). It was found that the proportion of GC content in STRs in the range 0–10 was maximum (68.75%) followed by the range 41–50 (15.32%) while the minimum was in the range 81–90 (0.002%) (Figure 4). Figure 5 shows the distribution of length of microsatellites in context to GC percentage. No correlation was found between size and GC content. Table 1 depicts the frequency of STRs based on their sizes. Maximum numbers were reported for the size ranging between 11–13 followed by the size 14–16. A comprehensive chromosome wise STR profile with its repeat type is depicted in Table 2.

**Table 1:** Frequencies of STRs based on their sizes

**Size of STRs**	**Number of STRs**	**Contribution in percentage**
<10	153185	16.82
11–13	276065	30.32
14–16	207831	22.83
17–25	162575	17.86
>25	110873	12.18

**Table 2:** Chromosome wise distribution of STRs

**Chromosomes**	**Simple**						**Compound**
	**Mono**	**Di**	**Tri**	**Tetra**	**Penta**	**Hexa**	
Chromosome 1	38690	12112	7672	454	1005	10	5858
Chromosome 2	35354	10911	7062	419	877	10	4899
Chromosome 3	32231	10406	6376	408	702	6	4611
Chromosome 4	30416	9552	6308	404	734	13	4382
Chromosome 5	22149	7486	4568	314	489	6	3365
Chromosome 6	20993	6941	4525	263	532	8	3132
Chromosome 7	21961	6884	4553	291	616	7	3282
Chromosome 8	22977	7190	4787	252	512	7	3333
Chromosome 9	20647	6278	4079	260	440	3	2937
Chromosome 10	20550	6425	4156	228	530	4	3082
Chromosome 11	19223	5662	3852	206	393	8	2701
Chromosome 12	19447	6285	3748	238	441	6	2725
Chromosome 13	17237	5220	3285	199	362	3	2721
Chromosome 14	15197	4703	2841	197	313	3	2162
Chromosome 15	14760	4892	2988	149	342	4	2219
Chromosome 16	15128	4630	3152	185	359	6	2353
Chromosome 17	14489	4585	2721	194	270	2	2160
Chromosome 18	11619	3814	2129	160	181	3	1746
Chromosome 19	13496	4373	2804	155	358	5	2098
Chromosome 20	12474	3949	2465	159	274	3	1832
Chromosome 21	11830	3467	2106	124	203	5	1588
Chromosome 22	12015	3893	2424	117	278	4	1705
Chromosome 23	18764	5898	3686	180	380	2	2704
Chromosome 24	7742	2571	1467	124	136	3	1127
Chromosome M	1	0	0	0	0	0	0
Chromosome U	50940	15373	9458	790	1279	26	9045
Chromosome X	15239	5241	3287	224	382	9	2704
Total	**535569**	**168741**	**106499**	**6694**	**12388**	**166**	**80471**

### STR validation

The previously published two sets of STR markers viz., heterologous [16] and homologous [17] were evaluated in the database using PERL script. The validated STRs are presented as positive primers in Table 3.

**Table 3:** STRs validation result of homologous and heterologous primer pairs of water buffalo

	**Heterologous**		**Homologous**	
	**ISAG–FAO recommended STRs from cattle**	**ISAG–FAO recommended STRs from buffalo**	**Nagarajan et al, monomorphic STRs from buffalo**	**Nagarajan et al, polymorphic STR loci from buffalo**
**Total no. of primer pairs reported**	30	30	7	107
**No. of positive primers (Forward)**	7 (23.33%)	7 (23.33%)	2 (28.57%)	49 (45.79%)
**No. of positive primers (Reverse)**	11 (36.67%)	8 (26.67%)	4 (57.14%)	37 (34.58%)
**No. of positive primers (common to both forward and reverse)**	3 (10.00%)	4 (13.33%)	2(28.57%)	26 (24.30%)

## Discussion and utility

A total of 910529 microsatellite markers have been searched by *in silico* mining. Simple STR were found to be most abundant (91.16%). Microsatellite density has been found positively correlated with genome size [18-20]. Among fully sequenced eukaryotic genomes, microsatellite density is highest in mammals. However in case of plant, microsatellite frequency is negatively correlated with genome size [21].

In the present study of water buffalo, mono- motif was found to be most abundant. Relative distributions of different microsatellite motif length classes in genomes differ considerably from species to species [22].

In case of water buffalo, it was found that longer repeats are less in abundance which is expected as reported and described in various studies [23,24]. It was also observed that microsatellite size range is increasing from 10 up to 14–16, however beyond this size range, it again starts decreasing. This is due to cyclical nature of microsatellite marker per say in its course of evolution. The birth of microsatellite starts with, out of register loop in event of DNA replication with a threshold size of 8 repeat unit or more, in the form of simple repeat. Gradually due to background mutation simple repeat gets converted in compound repeat. At the stage of simple repeat, the rate of mutation is high and predominantly it is addition of repeat unit and hence size increases. But once background mutation converts simple repeat into compound interrupted repeat, the smaller size simple repeat of less than 8 unit gets pinched off in subsequent replications. This maintains the size of microsatellite as evolutionary constraints otherwise microsatellite marker would have been always increasing in length during course of evolution. Thus individual microsatellites arrays have a “life cycle” of sorts, they are born, they grow and ultimately they perish. These events may stretch over tens or even hundreds of millions of years [25,26].

Water buffalo microsatellite profile exhibits the similar pattern. The relative abundance of repeat motif were in order of mono, di, tri, penta, tetra and hexa (Table 2). Though di-nucleotide repeats are most abundant in eukaryotic genome [27,28] but we found most abundance of mononucleotide repeats across all chromosome. This relatively higher abundance of mono over di nucleotide repeat type might be due to inherent limitation of the NGS technology which adds more mono nucleotide causing sequencing error [29]. The longer the chromosome proportionately higher the total repeat content as expected in ubiquitously distributed STR markers [30].

In order to validate the previously reported STR markers, two sets viz. heterologous (cattle original species and buffalo focal species), homologous STR (developed from buffalo and validated in buffalo) were considered. The heterologous markers recommended by FAO-IASG [16] and homologous marker [17] were used. It was observed that both subsets of heterologous ISAG-FAO recommended primer for cattle and buffalo diversity analysis gave less validation results i.e. 10% and 13.33% respectively. Cross species amplifiability is due to conservation of cattle STR and its flanking regions in other species [31]. Though some of the primers showed validation up to 36.67% (Table 3). In the cross species amplifiability of bovidae species, such data are usually expected due to null alleles and genomic changes during speciation [32]. In validation of homologous STR, it was found that both subsets reported higher percentage of monomorphic (28.57%) and polymorphic (24.30%) loci. The validation results are limited as the first draft genome assembly of buffalo is based on cattle and it is not completely finished.

The findings of this study has limitations which need to be addressed. As genome of water buffalo is just draft assembly based on cow assembly *Btau 4.0*, thus *de novo* assembly is needed to have the buffalo specific chromosome wise microsatellite profile. The current database is based on chromosome number of cattle which is certainly not the same in case of buffalo. For example cattle chromosome 4 is actually buffalo chromosome 8. In fact only chromosome number common between cattle and buffalo are just 5 viz 1, 2, 17, 18, and X [3]. The splitting and translocation has rendered syntenic relationship between these two species which are well documented. Nevertheless the microsatellites in our database with option of primer designing at desired place over “chromosome” will be of immense use especially over radiation hybrid of buffalo to resolve the problem and current issue of *de novo* assembly. Besides this, these markers can be further used for QTL, gene mapping as well as biodiversity analysis in setting the conservation priorities. The markers present in our database need further wet lab validation. Being first database of water buffalo microsatellite especially at juncture where *de novo* genome assembly is yet to be done, the use of these markers are highly warranted in order to “finishing” of water buffalo genome assembly. This will further lead to next version of buffalo microsatellite database base with proper buffalo specific chromosome wise data which is hitherto missing but critically needed. Such endeavour will fetch not only increase in buffalo productivity but also greater food security especially in third and new world countries.

## Conclusion

Being first buffalo STR database in the world, this would not only pave the way in resolving current water buffalo genome assembly problem but shall be of immense use for global community in QTL/gene mapping critically required to increase knowledge in the endeavour to increase buffalo productivity, especially for third world country where rural economy is significantly dependent on buffalo productivity.

## Availability and requirement

*BuffSatDb*, the buffalo microsatellite marker database is freely accessible for research purposes for non-profit and academic organizations at http://cabindb.iasri.res.in/buffsatdb/.

## Competing interests

The authors declare that they have no competing interests.

## Authors’ contributions

DK and AR conceived this study. S, VA & MAI created the work-flow, database, web-tool and performed data analyses. MAI, S, DK and AR drafted the manuscript. All authors read and approved the manuscript.
